# An Approach to the Examination of the Lumbar Plexus for Neurosurgical Residents: A Video Manuscript

**DOI:** 10.21315/mjms2024.31.1.19

**Published:** 2024-02-28

**Authors:** Nurul Ashikin Hamzah, Wei Lun Lee, Mohd Fakhri Md Fathil, Jafri Malin Abdullah, Zamzuri Idris, Abdul Rahman Izaini Ghani, Sanihah Abdul Halim

**Affiliations:** 1 Department of Neurosciences, School of Medical Sciences, Universiti Sains Malaysia Kelantan, Malaysia; 2 Brain and Behaviour Cluster, School of Medical Sciences, Universiti Sains Malaysia, Kelantan, Malaysia; 3 Department of Neurosciences & Brain and Behaviour Cluster, Hospital Universiti Sains Malaysia, Universiti Sains Malaysia, Kelantan, Malaysia; 4 Department of Neurosurgery, Hospital Sultanah Nur Zahirah, Terengganu, Malaysia; 5 Department of Neurosurgery, Hospital Kuala Lumpur, Kuala Lumpur, Malaysia; 6 Department of Neurosurgery, Sarawak General Hospital, Sarawak, Malaysia

**Keywords:** lumbar plexus, lower limb examination, neurology, neurosurgery

## Abstract

The lumbar plexus provides innervation to the lower limbs and is essential in enabling motor movement and sensation in the lower limbs. Some of its branches also innervate the muscles in the pelvic girdle. Compared to the brachial plexus in the upper limbs, the lumbar plexus appears to garner less recognition among physicians and surgeons. However, it is important to understand the anatomy of the lumbar plexus and its branches along with the innervation they enable, as injury to them can cause plexopathies and pathologies that should be recognised by any treating clinician. Lumbar disc herniation, trauma and entrapment by muscles or hypertrophic ligaments are common causes of lumbar plexus or nerve injuries. A video was produced to demonstrate the examination techniques explained in this article. To provide comprehensive examination of the lower limbs, the sciatic nerve and its branches are also included in the examination video.

## Introduction

The examination of the lumbar plexus is underrepresented compared to that of its counterpart, the brachial plexus. This paper will present a new approach to the examination of the lumbar plexus. A link to a video demonstrating this approach is provided at the end of the article.

### Anatomy of the Lumbar Plexus

The T12–L4 nerve roots are the main source of the lumbar plexus’s branches (1). The lumbar plexus is located in the retroperitoneum, below and inside the psoas muscle. Thus, pathologies within the retroperitoneum, such as haematomas or abscesses, are a common cause of lumbar plexopathy (2). The iliohypogastric (L1), ilioinguinal (L1), genitofemoral (L1–L2) and lateral cutaneous nerves in the thigh (L2–L3) are primarily responding for enabling sensory input in the lower limbs.

The rootlets from L2 to L4 comprise the anterior and posterior divisions of the lumbar plexus. The anterior division forms the obturator nerve, which innervates the adductor muscles in the thigh and the skin in the medial thigh. The posterior division forms the femoral nerve, from which motor branches extend to innervate the psoas muscle, the iliacus muscle and pectineus muscle prior to entering the upper leg.

In the upper leg, the anterior division extends sensory branches to the medial part of the thigh via the medial cutaneous and intermediate cutaneous nerves in the thigh. The posterior division of the femoral nerve then proceeds to supply the quadriceps muscles before producing the terminal branch, called the saphenous nerves, which provide sensory supply to the skin of the medial side of the leg and foot.

**Figure 1:**
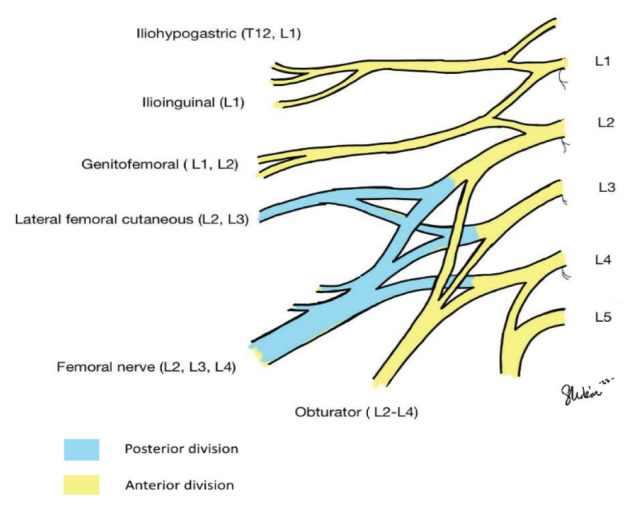
The lumbar plexus

**Figure 2:**
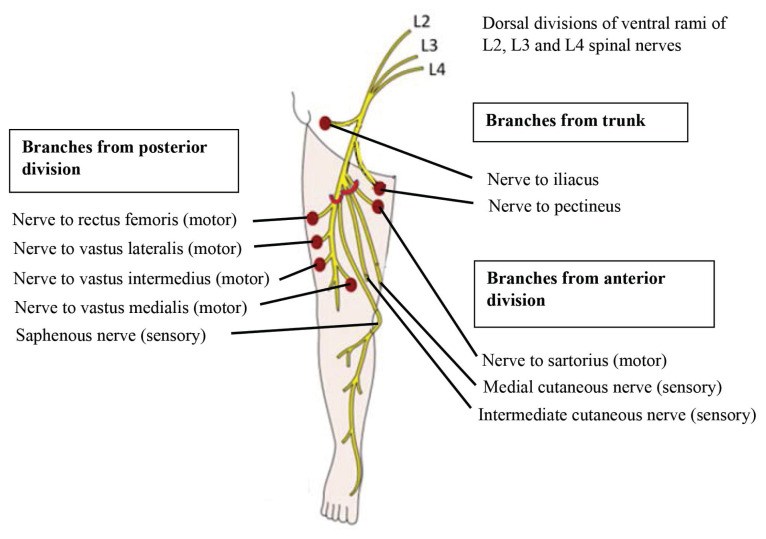
Branches of femoral nerve

The medial and intermediate cutaneous nerves in the thigh and saphenous nerves, respectively, innervate the quadriceps muscle after they pass beneath the inguinal ligament and supply feeling to the medial thigh and lower leg. The obturator nerve is formed by contributions from L2–L4, while the femoral nerve is formed by the posterior division of these contributions. The medial thigh skin and adductor muscles in the thigh are both innervated by the obturator nerve. The psoas and iliacus muscles are proximally innervated by the femoral nerve. Figure 1 illustrates the nerve roots and its division, while Figure 2 illustrates the branches of the femoral nerve. Table 1 shows the anatomy of the lumbar plexus, the supply, it’s innervation and it’s clinical correlation. Table 2 will show clinical signs and symptoms in relation to dorsal root ganglia.

### Examination Approach

#### Inspection

Both lower limbs must be properly exposed and in a natural physiological position to assess the attitude of the limbs and observe muscle bulk, atrophy, asymmetry, scars and signs of injury (3). Each limb must be compared to the other during every step of the examination.

#### Muscle Tone

Muscle tone is defined as ‘the tension in the relaxed muscle’ or ‘the resistance, felt by the examiner during passive stretching of a joint when the muscles are at rest’ (4). Meanwhile, spasticity was described by Lance JW in 1980 as ‘a motor condition characterised by a velocity dependent increase in tonic stretch reflexes (muscle tone) with accentuated tendon jerks.’ During assessments of tone, patients need to be relaxed and cooperative. Small talk may help.

Muscle tone can be graded using O’Sullivan’s scale:

0: No response
1+: Decreased response (hypotonia)
2+: Normal
3+: Exaggerated response (mild to moderate hypertonia)
4+: Sustained response (severe hypertonia)

Spasticity can be graded using the modified Ashworth scale (5):

0: No increase in muscle tone
1: Slight increase in muscle tone, manifested by a catch and release or by minimal resistance at the end of the range of motion (ROM), when the affected part is moved in flexion or extension
1+: Slight increase in muscle tone, manifested by a catch followed by minimal resistance throughout the remainder (less than half) of the ROM
2: More marked increase in muscle tone throughout most of the ROM, but the affected part is easily moved
3: Considerable increase in muscle tone, passive movement difficult
4: The affected part is rigid in flexion or extension

#### Motor Examination

Each muscle should be tested (as per Table 3) and graded using the modified Medical Research Council scale:

0: No contraction
1: A flicker or trace of contraction
2: Active movement with gravity eliminated
3−: Muscle moves the joint against gravity, but not through full mechanical ROM
3: Muscle moves the joint against gravity but cannot hold joints against resistance
3+: Muscle moves the joint against gravity and is capable of transient resistance but collapses abruptly
4−: Active movement against gravity, able to hold against minimal resistance
4: Active movement against gravity, able to hold against moderate resistance
4+: Active movement against gravity, able to hold against moderate to maximal resistance
5−: Barely detectable weakness
5: Normal power

#### Reflex

Reflex is an involuntary response to a sensory stimulus. It has afferent and efferent components.

- Cresmatic reflexes (ilioinguinal and genitofemoral nerves, L1–L2 and cutaneous reflex), can be assessed by lightly scratching or pinching the skin on the upper, inner aspect of the thigh. The expected response is a contraction of the cresmatic muscle with a quick elevation of the ipsilateral testicle. This can be absent in elderly males or individuals with hydrocele, torsion, orchitis or epididymitis.
- Gluteal reflex (inferior gluteal nerve, L4, S2 and cutaneous reflex) - a contraction of the gluteal maximus muscle after stroking the skin over the buttock.
- Patellar reflex (femoral nerve, L3–L4 and deep tendon reflex) - a contraction of the quadriceps resulting in extension of the knee.
- Adductor reflex (L2–L4 and deep tendon reflex) - with the thigh in slight abduction, tap either the medial epicondyle of the femur in the vicinity of the adductor tubercle or the medial condyle of the tibia, resulting in contraction of the adductor muscle of the thigh and inward movement of the limb.
- Medial hamstring reflex (L5 and deep tendon reflex) - the semitendinosus and semimembranosus muscles are struck; the leg is abducted and slightly externally rotated, and the knee is flexed. The fingers are placed over the tendon on the medial posterior aspect of the knee and tapped with a reflex hammer to observe the contraction of the muscle.

#### Sensory Examination

**Figure 3:**
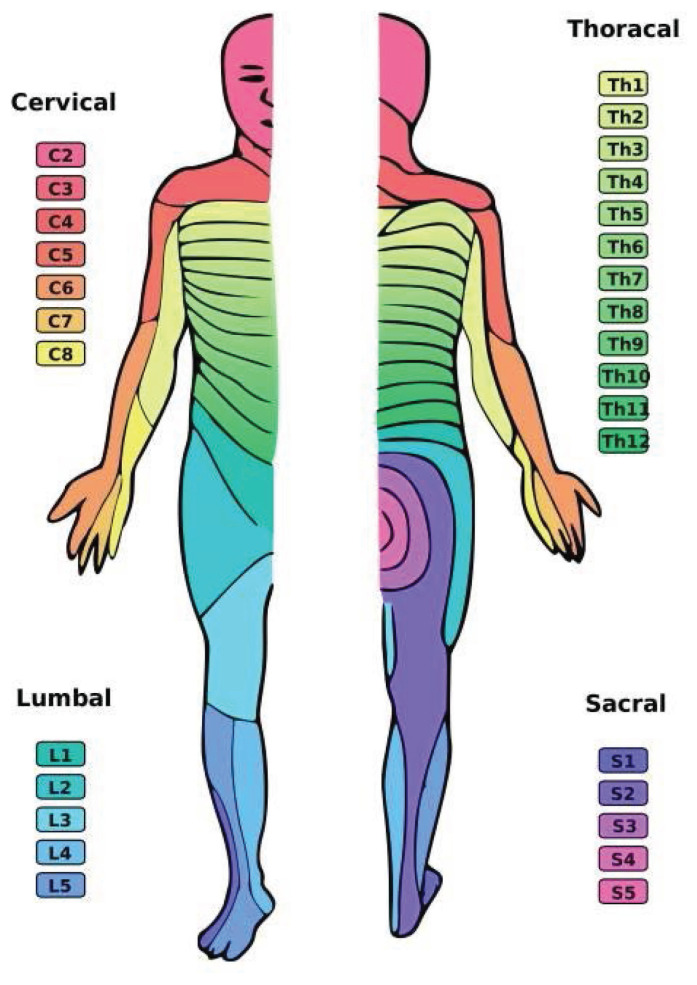
Sensory dermatomes (6)

Sensation - such as a light touch, pain, temperature, vibration, 2-point discrimination, kinaesthesia, proprioception or stereognosis - can be assessed by localising the lesion, i.e. at the level of the spinal nerves, spinal cord or cortical. The sensory level then can be graded and charted using the American Spinal Injury Association (ASIA) chart (7) according to the relevant dermatome, refer to Figure 3. Moreover, in the examination of peripheral nerves, stocking-glove distribution also needs to be examined, especially in cases of diabetic neuropathy.

Grading scale for sensation:

0: Absent, no response
1: Decreased, delayed response
2: Increased, exaggerated response
3: Inconsistent response
4: Intact, normal response
  NT: Unable to test
  P = Proximal; D = Distal

#### Autonomic System

The lumbar plexus is partly involved in bladder function. It supplies the internal urethral sphincter at the neck of the bladder. It is supplied by the intermediolateral column at the T12–1L1 level via the sympathetic prevertebral plexus and hypogastric nerve. If any lesion affects the sympathetic ganglia, the function of this muscle will be affected, causing urinary loss (1). Any reduced sweating of the nerve distribution also needs to be taken into consideration during examination.

Together with this article, attached the link to the video of examination of lumbar plexus examination, which lasted for 15 min.

YouTube link for lumbar plexus examination: https://youtu.be/2IdLe2i-Zxc

## Conclusion

We hope that this comprehensive evaluation of the lumbar plexus will be helpful in localising and identifying the extent of injuries that patients may have.

## Figures and Tables

**Table 1:** Anatomy of lumbar plexus and its clinical correlations

Nerve	Supply	Signs and symptoms of injury	Note
Iliohypogastric nerve (L1)	Sensory: skin over the gluteal region and hypogastric region, just above the symphysis pubis	Sensory loss over the distribution area	
Ilioinguinal nerve (L1)	Sensory: skin of the upper and medial thigh; upper part of the root of the penis and the scrotum in the male; mons pubis and labia majora in the female	Sensory loss over the distribution area	
Genitofemoral nerve (L1–L2)	Motor: cremaster muscle Sensory: skin of the scrotum, labia, small area of upper thigh	Sensory loss over the supply region, loss of cresmasteric reflexes	Testicular torsion may entrap the genital branch
Lateral femoral cutaneous nerve (L2–L3)	Sensory: anterolateral aspect of the thigh	Sensory loss over the distribution area *Meralgia paraesthesia, a clinical syndrome characterised by itching, burning, pain and numbness over the anterolateral aspects of the thigh	Nerve entrapment, where it passes under or through the inguinal ligament medial to the anterior superior iliac spine or pierces the fascia lata Usually occurs due to iatrogenesis, i.e. skin incision, transection, suture ligation, belt braces, accidental trauma
Obturator nerve (anterior division of L2–L4)	Motor: Adductor muscle of thigh, gracilis, obturator externus Sensory: small area of medial aspect of thigh	Weakness of adduction and external rotation of thigh Anaesthesia over inner surface of thigh	
Femoral nerve (posterior division of L2–L4)	Motor: supplies psoas, iliacus, sartorius, pectineus and quadriceps muscle Sensory: intermediate (anterior) and medial femoral cutaneous nerve, innervates the skin of the anterior thigh Extensive cutaneous field along medial aspect of lower leg and medial aspect of the foot	Impairment of knee extension Difficulty in walking forward, climbing stairs Weakness in hip flexion Patellar reflex impaired Sensory loss over anterior and medial aspect of thigh and medial aspect of leg	Vulnerable to injury in the lower abdomen and upper thigh due to its superficial course in the region and proximity to the acetabular rim Injury to the pelvis: Results in ipsilateral weakness in hip flexion and knee extension; sensory impairment of anterior thigh and medial aspect of leg and foot; loss of knee-jerk reflex; atrophy of the quadriceps with loss of patellar reflex Injury within thigh: Range of clinical presentation; complete or isolated motor or sensory deficit Injury to saphenous nerve: Sensory disturbances over medial aspect of the leg extending along the inner border of the foot to the big toe

**Table 2:** Clinical signs and symptoms in relation to dorsal root ganglia

	Preganglionic	Postganglionic
Site	Injury between the rootlet and dorsal root ganglia	Injury distal to dorsal root ganglia
Sensory loss	Present - may involve one or more nerve	Present - limited to the distribution of the affected nerve
Motor	Lower motor neuron-type muscle weakness may not be apparent, as the muscles in the lower limb are supplied by one or more root	Lower motor neuron-type weakness, atrophy
Reflex	Absent	Absent
Autonomic	Affected - reduced sweating in the distribution region Internal urethral sphincter muscle affected - causes the muscle to continuously relax, thus unable to contain urine	Mostly intact

**Table 3:** Motor examination of lumbar plexus

Muscles	Nerve and nerve roots	Functions	Examination
Iliopsoas (gracilis, iliacus, psoas)	Spinal nerve (L1, L2 and L3) and femoral nerve (L1, L2 and L3)	Flexes leg at hip	Patient flexes the thigh at the hip against resistance, flexes the knee and hip with the leg
Quadriceps femoris (rectus femoris, vastus lateralis, vastus intermedius, vastus medialis)	Femoral nerve (L2, L3 and L4)	Extends leg at knee	Patient extends the leg against resistance with the limb flexed at the hip and the knee To detect slight weakness, the leg should be fully flexed at knee
Adductors (obturator externus, adductor longus, adductor magnus, adductor brevis)	Obturator nerve (L2, L3 and L4)	Adducts and outwardly rotates leg (obturator externus), adducts thigh (adductor longus, magnus and brevis)	Patient lies on back with leg extended at the knee and adducts the limb against resistance
Gluteus medius and minimum	Superior gluteal nerve (L4, L5 and S1)	Abducts and medially rotates thigh	Patient lies on back and internally rotates the thigh against resistance with the limb flexed at the hip and knee
Gluteus medius and minimus and tensor fasciae lata	Superior gluteal nerve (L5 and S1)	Abducts and medially rotates thigh	Patient lies on back with leg extended and abducts the limb against resistance
Gluteus maximus	Inferior gluteal nerve (L5, S1and S2)	Extends, abducts and laterally rotates thigh; extends lower trunk	Patient lies on back with leg extended at the knee and extends the limb at the hip against resistance
Hamstring muscles (adductor magnus, semitendinosus, semimembranosus, biceps femoris)	Sciatic nerve (L5, S1 and S2)	Adducts thigh (adductor magnus), flexes knee, medially rotates thigh, extends hip (semitendinosus, semimembranosus), flexes knee, extends hip (biceps femoris)	Patient lies on back with the limb flexed at the hip and knee, and flexes the leg at the knee against resistance
Hamstring muscles (adductor magnus, semitendinosus, semimembranosus, biceps femoris)	Sciatic nerve (L5, S1 and S2)	Adducts thigh (adductor magnus), flexes knee, medially rotates thigh, extends hip (semitendinosus, semimembranosus), flexes knee, extends hip (biceps femoris)	Patient lies on face and flexes leg at the knee against resistance
Gastrocnemius	Tibial nerve (S1 and S2)	Plantar flexes foot	Patient lies on back with leg extended and plantar-flexes foot against resistance To detect slight weakness, the patient should be asked to stand on one foot, raise the heel from the ground and maintain the position
Soleus	Tibial nerve (S1 and S2)	Plantar-flexes foot	Patient lies on back with limb flexed at the hip and knee and plantar-flexes the foot against resistance
Tibialis posterior	Tibial nerve (L4, and L5)	Plantar flexes and inverts foot	Patient inverts foot against resistance
Flexor digitorum longus, flexor hallucis longus	Tibial nerve (L5, S1 and S2)	Flexes distal phalanges, aids plantar flexion	Patient flexes toes against resistance
Tibialis anterior	Deep peroneal nerve (L4 and L5)	Dorsiflexes and inverts foot	Patient dorsiflexes the foot against resistance
Extensor digitorum longus	Deep peroneal nerve (L5 and S1)	Extends phalanges, dorsiflexes foot	Patient dorsiflexes phalanges against resistance
Extensor hallucis longus	Deep peroneal nerve (L5 and S1)	Extends great toe, aids dorsiflexion	Patient dorsiflexes distal phalanx of big toe against resistance
Extensor digitorum brevis	Deep peroneal nerve (L5 and S1)	Extends toes	Patient dorsiflexes proximal phalanges of toes against resistance
Peroneus longus and brevis	Superficial Peroneal nerve (L5 and S1)	Plantar flexes and everts foot	Patient everts foot against resistance
Small muscles of the foot	Medial and lateral plantar nerves (S1 and S2)	Cup sole	Patient cups the sole of their foot
